# Tight regulation of wingless-type signaling in the articular cartilage - subchondral bone biomechanical unit: transcriptomics in *Frzb*-knockout mice

**DOI:** 10.1186/ar3695

**Published:** 2012-01-20

**Authors:** Liesbet Lodewyckx, Frédéric Cailotto, Sarah Thysen, Frank P Luyten, Rik J Lories

**Affiliations:** 1 Laboratory for Skeletal Development and Joint Disorders, Department of Development and Regeneration, KU Leuven, Belgium; 2 Division of Rheumatology, University Hospitals Leuven, Belgium

## Abstract

**Introduction:**

The aim of this research was to study molecular changes in the articular cartilage and subchondral bone of the tibial plateau from mice deficient in frizzled-related protein (Frzb) compared to wild-type mice by transcriptome analysis.

**Methods:**

Gene-expression analysis of the articular cartilage and subchondral bone of three wild-type and three *Frzb^-/- ^*mice was performed by microarray. Data from three wild-type and two *Frzb^-/- ^*samples could be used for pathway analysis of differentially expressed genes and were explored with PANTHER, DAVID and GSEA bioinformatics tools. Activation of the wingless-type (WNT) pathway was analysed using Western blot. The effects of *Frzb *gain and loss of function on chondrogenesis and cell proliferation was examined using ATDC5 micro-masses and mouse ribcage chondrocytes.

**Results:**

Extracellular matrix-associated integrin and cadherin pathways, as well as WNT pathway genes were up-regulated in *Frzb^-/- ^*samples. Several WNT receptors, target genes and other antagonists were up-regulated, but no difference in active β-catenin was found. Analysis of ATDC5 cell micro-masses overexpressing *FRZB *indicated an up-regulation of *aggrecan *and *Col2a1*, and down-regulation of molecules related to damage and repair in cartilage, *Col3a1 *and *Col5a1*. Silencing of *Frzb *resulted in down-regulation of *aggrecan *and *Col2a1*. Pathways associated with cell cycle were down-regulated in this transcriptome analysis. Ribcage chondrocytes derived from *Frzb^-/- ^*mice showed decreased proliferation compared to wild-type cells.

**Conclusions:**

Our analysis provides evidence for tight regulation of WNT signalling, shifts in extracellular matrix components and effects on cell proliferation and differentiation in the articular cartilage - subchondral bone unit in *Frzb^-/- ^*mice. These data further support an important role for FRZB in joint homeostasis and highlight the complex biology of WNT signaling in the joint.

## Introduction

Homeostasis of articular cartilage and subchondral bone is essential for maintenance of joint function which is critically dependent on the balance between anabolic and catabolic signaling pathways [1,2]. It requires maintenance of the stable phenotype that characterises the articular cartilage, sustained extracellular matrix (ECM) synthesis, efficient breakdown and clearance of damaged macromolecules and dead cells, as well as functional and molecular adaptations to mechanic loads. Loss of homeostasis results in gradual deterioration of cartilage quality and thickening of the subchondral bone, progressively leading to osteoarthritis (OA).

The wingless-type (WNT) signaling pathway plays an important role in cartilage, bone and joint development and has been associated with postnatal joint homeostasis and disease [3,4]. WNTs are a group of at least 19 structurally related secreted glycoproteins that activate different intracellular cascades [5]. Among these, canonical WNT signaling involving β-catenin has been studied best. In the absence of a WNT-Frizzled low density lipoprotein receptor-related protein-5/6 co-receptor interaction (WNT-FZD-LRP5/6), β-catenin is caught in a molecular destruction complex, phosphorylated and degraded by the proteasome. Upon WNT-receptor interaction, the destruction complex is disassembled, β-catenin accumulates in the cell, translocates to the nucleus and associates with transcription factors of the T-cell factor/lymphoid enhancer factor (TCF/LEF) family. Alternatively, non-canonical WNT signaling can alter calcium balances in the cell or activate protein kinases [5,6].

WNTs, their extracellular antagonists, such as the secreted frizzled-related proteins (SFRPs), co-receptor inhibitors, such as the dickkopfs (DKKs), and β-catenin have been studied in animal models of OA and OA patients [7-13]. Current data suggest that canonical WNT signaling plays an essential role in joint and bone formation [14,15] and in the maintenance of the articular cartilage phenotype, which is characterised by extended cell survival and absence of differentiation towards hypertrophy [16]. Cartilage-specific inhibition of β-catenin results in an OA-like phenotype with chondrocyte apoptosis [8]. Cartilage-specific overexpression of a constitutively active form of β-catenin also results in an OA-like phenotype, but here the disease is characterised by loss of the chondrocyte's differentiation status and expression of hypertrophic markers [9].

Frizzled-related protein (Frzb, also known as SFRP3) is a WNT antagonist originally identified from a chondrogenic extract of articular cartilage [17] and plays a role in skeletal development [17,18]. Polymorphisms in *FRZB *have been associated with OA [3]. We previously developed mice that are genetically deficient in *Frzb*. These mice do not develop spontaneous arthritis but are more susceptible to OA in induced models [7]. This observation has been linked to increased WNT signaling and *Mmp3 *expression in the articular cartilage. The cortical bone in these mice is thicker and the bones show an enhanced anabolic response upon mechanical loading compared to wild-type mice. In this study, we used *Frzb^-/- ^*mice to further evaluate how the absence of a WNT antagonist affects molecular homeostasis in the articular cartilage and subchondral bone.

## Materials and methods

### Mice and tissue sampling

*Frzb*^-/- ^mice were generated in our research group [7] and back-crossed into the C57Bl/6J background for over 10 generations. Genotypes were determined as described [7]. Six-week-old male *Frzb*^-/- ^and wild-type mice were sacrificed by cervical dislocation. The articular cartilage and subchondral bone from the tibial plateau of the knee joint of the hind limb was carefully dissected in one piece at the growth plate region using micro-dissection forceps, a procedure easy to perform at this age when the growth plate is not yet closed. The tissues were immediately snap frozen in liquid nitrogen and stored at -80°C until further processing or used for histology. Animal procedures were approved by the Ethical Committee for Animal Research, KULeuven.

### Microarray hybridization and data acquisition

Per microarray, articular cartilage and subchondral bone from a single joint were used. Samples were homogenised using the Fastprep-24 tissue-homogeniser (MP Biomedicals, Solon, OH, USA) in lysing matrix A tubes and RLT lysis buffer (RNeasy Fibrous Tissue kit (Qiagen, Chatsworth, CA, USA)). Samples were kept under pre-cooled conditions using the CryoPrep Adaptor. RNA was isolated with the RNeasy Fibrous Tissue kit (Qiagen) with proteinase K and deoxyribonuclease (DNaseI) treatment. RNA concentration and purity were assessed with a NanoDrop Spectrophotometer (NanoDrop Technologies, Centreville, DE, USA) and integrity was determined using RNA nanochips and the Agilent 2100 Bio-analyzer (Agilent Technologies, Diegem, Belgium). Only non-degraded RNA without impurities (RNA integrity number > 7.7), was considered for microarray analysis.

Transcriptional profiles of three *Frzb^-/- ^*and three wild-type samples were analyzed by the VIB Microarray Facility [19]. Per sample, 2 μg of total RNA spiked with bacterial RNA transcript positive controls (Affymetrix, Santa Clara, CA, USA) was converted to double stranded cDNA. Subsequently, the sample was converted and amplified to antisense cRNA and labeled with biotin. A mixture of purified and fragmented biotinylated cRNA and hybridisation controls (Affymetrix) was hybridised on Affymetrix GeneChip Mouse Genome 430-2.0 arrays followed by staining and washing in a GeneChip fluidics station 450 (Affymetrix). To assess the raw probe signal intensities, chips were scanned using a GeneChip scanner 3000 (Affymetrix). Microarray data have been deposited in the Gene Expression Omnibus (GEO) [20] and are accessible through Gene Expression Omnibus accession number GSE33656.

### Western blot analysis

Proteins were isolated from the dissected articular cartilage and subchondral bone pieces using cell extraction buffer (Invitrogen, Merelbeke, Belgium) supplemented with 1 mM phenylmethanesulfonyl (Sigma-Aldrich, Bornem, Belgium) and 5% protease inhibitor cocktail (Sigma-Aldrich) using the Fastprep-24 tissue homogeniser (MP Biomedicals). A total of 20 μg of each sample was denatured and separated on a 4 to 12% polyacrylamide Bis-Tris gel (Invitrogen) by electrophoresis using NuPage MES SDS Running buffer (Invitrogen). Proteins were transferred to a PVDF (polyvinylidene difluoride) membrane (Millipore, Brussels, Belgium). Non-specific binding sites were blocked using 5% blottoB (Santa Cruz Biotechnology, Santa Cruz, CA, USA) in Tris-buffered saline with 0.1% Tween (TBS/T) for one hour at room temperature. Blots were probed overnight at 4°C with the following antibodies: 1/500 dephospho-β-catenin (CTNNB1) sheep antibody (Genway Biotech, San Diego, CA, USA), 1/1,000 phospho-Smad1(Ser463/465)/Smad5(Ser463/465)/Smad8(Ser426/428) rabbit antibody (Cell Signaling Technology, Danvers, MA, USA), 1/500 anti-SFRP1 rabbit antibody (Abcam, Cambridge, UK), 1/500 mouse DKK2 affinity purified polyclonal goat antibody, 1/1,000 mouse SFRP2 affinity purified polyclonal goat antibody (both from R&D Systems, Minneapolis, MN, USA) or 1/4,000 anti-GAPDH mouse monoclonal 6C5 (Ambion, Applied Biosystems) in 5% bovine serum albumin in TBS/T with 0.02% sodiumazide. Horseradish peroxidase-conjugated donkey anti-sheep (1/5,000), mouse anti-rabbit (light chain specific) (1/5,000), donkey anti-goat (1/5,000) and goat anti-mouse (1/50,000) polyclonal antibodies (Jackson ImmunoResearch Laboratories, West Grove, PA, USA) in 5% blottoB in TBS/T were used as secondary antibodies. Blots were visualised using Western Lightning Chemiluminescent Substrate (Perkin Elmer Life and Analytical Sciences, Inc., Waltham, MA, USA) for dephospho-β-catenin, DKK2, SFRP2, SFRP1 and GAPDH or SuperSignal West Femto Maximum Sensitivity Substrate (Pierce, Thermo Scientific, Rockford, IL, USA) for phosphorylated Smad. Densitometry analysis was performed with ImageJ Software (NIH Image, National Institutes of Health, Bethesda, MD, USA [21]).

### Cell culture experiments

ATDC5 cells were cultured in maintenance medium (1:1 Dulbecco's modified Eagle's medium (DMEM):Ham's F-12 mix (Gibco Life Technologies, Gent, Belgium), 1% antibiotic-antimycotic (AB) (Gibco), 5% fetal bovine serum (FBS) (Gibco) containing 10 μg/ml human transferrin and 30 mM sodiumselenite (Sigma-Aldrich) and maintained in a humidified atmosphere of 5% CO_2 _and 95% O_2 _at 37°C.

In *FRZB *overexpression experiments, ATDC5 cells were transfected with control pcDNA3.1+ (Invitrogen) or the pcDNA3.1-full length *FRZB *construct (pfrzb [17]) using lipid-based agent Fugene HD (Roche Diagnostics, Vilvoorde, Belgium). After 24 hours, selection with 1 mg/ml geneticin (Gibco) was initiated. Selection medium was renewed every day for 14 days. Antibiotic resistant cells were dilution-cloned.

In *Frzb *knock-down experiments, ATDC5 cells were transfected with control pGIPZ-non-silencing shRNAmir (Open Biosystems, Thermo Scientific IT IS Open Biosystems, Thermo Scientific, Lafayette, CO, USA) or with a pGIPZ-shRNAmir directed against *Frzb *(Open Biosystems) using lipo-polymeric agent Arrest-In (Open Biosystems). After 24 hours, selection with 0.5 μg/ml puromycin was initiated. Selection medium was renewed every day for seven days. Antibiotic resistant cells were dilution-cloned.

Stably-transfected ATDC5 cells were grown in micro-masses to undergo chondrogenesis. Three drops cell suspension (2 × 10^5 ^cells) were placed in a single well of a standard 12-well culture plate. The cells were allowed to adhere for two hours at 37°C, then 1 ml maintenance medium was added to each well. Geneticin or puromycin pressure was maintained during chondrogenesis.

Micro-masses were cultured in the maintenance medium containing an ITS premix (10 μg/ml insulin, 5 μg/ml human transferrin and 30 mM sodiumselenite) (Gibco) and 5 μg/ml human transferrin for two weeks. The mineralization phase was induced using α-MEM medium (Gibco) containing 5% fetal bovine serum (Gibco), ITS premix, 5 μg/ml human transferrin and 7 mM beta-glycerolphosphate (Sigma-Aldrich) from Day 14 until Day 21. Each condition was performed in triplicate. Total RNA from micro-masses was isolated after 7, 14 or 21 days in culture using the Nucleospin RNA II kit (Macherey-Nagel, Düren, Germany).

Protein extraction of the micro-masses stably overexpressing *FRZB *or controls after seven days was performed using cell extraction buffer supplemented with 1 mM phenylmethanesulfonyl and 5% protease inhibitor cocktail, followed by quantification using the Pierce BCA Protein Assay kit (Thermo Scientific).

Some ATDC5 micro-masses were fixed in 95% ice-cold methanol for staining. For Picrosirius Red, micro-masses were stained for one hour in Picrosirius Red (0.1% Direct Red 80 (Sigma-Aldrich) in a saturated aqueous solution of picric acid), washed three times with 0.5% acetic acid in water and air-dried. For Safranin O, micro-masses were stained for one hour in Safranin O (1% alcoholic solution (Klinipath, Olen, Belgium)), washed three times with water and air-dried. Quantification of the staining was performed by dissolving the micro-masses with 1 M NaOH (for Picrosirius Red) or 6M Guanidine-HCl (for Safranin O) (both from Sigma-Aldrich) and by measuring the absorbance at 540 and 512 nm respectively with the Infinite M200 (Tecan, Männedorf, Switzerland).

### cDNA synthesis and Quantitative Real-Time PCR

Complementary DNA was synthesised from 1 μg of RNA isolated from tibia articular cartilage and subchondral bone pieces or ATDC5 cell micro-masses using the RevertAid H minus First Strand cDNA synthesis kit (Fermentas GmbH, St-Leon-Rot, Germany). TaqMan gene expression assays (Applied Biosystems, Carlsbad, CA, USA) or the SYBRgreen master mix system (Fermentas) were used to verify differential expression of *Frzb *(Mm00441378_m1), *Sfrp1 *(Mm00489161_m1), *Sfrp2 *(Mm0485986_m1), *Dkk2 *(Mm00445025_m1), *aggrecan *(forward 5'-GCTGCAGTGATCTCAGAAGAAG-3', reverse 3'-GATGGTGAGGGAAGACCCTA-5'), *Col3a1 *(forward 5'-TTATTCTCCCCAATTCGACTCA-3', reverse 3'-AGATCCAGGATGTCCAGAAGAA-5'), *Col5a1 *(forward 5'-CGGATGTTGCCTACCGAGT, reverse 3'-ACGGTTGTCAGGATGGAGAA-5') and *Col2a1 *(Mm01309565_m1) (forward 5'-CCAGGATGCCCGAAAATTAG-3', reverse 3'-TTCTCCCTTGTCACCACGAT-5'). For TaqMan assays analysis was performed using the PerfeCTa qPCR FastMix UNG (Quanta Biosciences, Gaithersburg, MD, USA) using the following conditions: 1 minute at 95°C, 40 cycles of 3 seconds of denaturation at 95°C, followed by 20 seconds of annealing-extension at 60°C. All experiments were performed in duplicate. For SYBRgreen, quantitative analysis was performed as follows: 10 minutes at 95°C, 40 cycles of 15 seconds of denaturation at 95°C, followed by 60 seconds of annealing-extension at 60°C. Melting curve analysis was performed to ensure amplification of a specific product. The Corbett Rotor-Gene 6000 (Corbett Research, Westburg, Leusden, The Netherlands) was used for both systems. Results are expressed using the comparative threshold method [22] and were normalised to housekeeping gene *Hprt *(hypoxanthine guanine phosphoribosyl transferase) (Mm00446968_m1 or forward 5'-TGCTGACCTGCTGGATTACA-3', reverse 3'-TATGTCCCCCGTTGACTGAT-5').

### Mouse rib chondrocyte isolation and proliferation analysis

Rib and sternum chondrocytes were isolated from three six-week-old wild-type and three *Frzb^-/- ^*mice, as described with minor modifications [23]. The sternum was longitudinally cut, followed by complete removal of the ventral part of the ribcage. The ribcage was washed three times in Dulbecco's phosphate buffered saline (DPBS) (Lonza, Verviers, Belgium) with 1% AB (Gibco). Soft tissues were digested in 3 mg/ml collagenase D (Roche Diagnostics) in medium (DMEM, 1% AB and 1% sodium pyruvate (Invitrogen)) for 1 h standing upright in a collection tube in humidified atmosphere of 5% CO_2 _and 95% O_2 _at 37°C, followed by rotation for a further 1.5 h. Soft tissues were carefully removed, followed by further digestion in fresh 3 mg/ml collagenase D in medium when the soft tissues kept adhering. After washing twice in DPBS with 1% AB, cartilage was digested using 1 mg/ml collagenase D in medium overnight in a petri dish in the incubator. The medium containing chondrocytes was transferred to a collection tube. The bones were rinsed with complete growth medium (10% FBS (Gibco)) and this was also transferred to the collection tube. After centrifugation, cells were resuspended in 4 ml complete growth medium, plated on a T25 plate (Greiner Bio-One, Frickenhausen, Germany) and grown until confluent. The medium was changed every two days. For the proliferation assay, chondrocytes from three *Frzb*^-/- ^and three wild-type mice were plated at different cell densities (500, 2,000 or 4,000 cells/well) in triplicate on fluorescence compatible 96-well flat bottom plates (μClear-plate, black, 98-well, Greiner Bio-one). Fluorescence was measured 24 h and 1 week after plating using the CyQuant NF Cell proliferation kit (Molecular Probes, Invitrogen) and the Wallac Victor 1420 Multilabel counter (Perkin Elmer) at an excitation wavelength of 485 nm and emission of 535 nm. The difference in fluorescence between the two time points (24 h and 1 week) was calculated and considered the amount of proliferation in that time window. A different plate was used for each time point.

### Bioinformatics analysis and statistics

The quality of hybridization and data acquisition was assessed by RNA-degradation plots, histograms of the perfect match values distribution and quality control graphs. Data were pre-processed by removal of the hybridisation, labeling control and absent probe sets, followed by a log2 transformation and normalisation of the results to obtain the Robust Multiarray Averaging (RMA) algorithm defined expression values and the Microarray Analysis Suite (MAS) 5.0 software detection calls. Significant differences in gene expression were defined using a modified t-test by the limma package from Bioconductor [24] followed by Benjamini-Hochberg multiple testing correction. For further analysis, we used the PANTHER [25], DAVID [26] and GSEA [27] tools [28-33].

PANTHER uses pathways compiled by experts and determines the representation of a specific pathway on the selected gene list by applying a binomial statistic to which we applied an additional false discovery rate (FDR) test. Only pathways that included at least 15 annotated genes were taken into consideration. With DAVID we interrogated representation in KEGG [34] and Biocarta pathways [35]. It uses a modified Fisher's exact test and applies a Benjamini-Hochberg multiple testing correction. The GSEA system uses all data in the microarray analysis in a ranked list and compares a maximal enrichment score to a series of 1,000 random permutations resulting in nominal *P*-values and FDR q-values. For GSEA analysis, the KEGG curated pathway set, the miRNA motif and transcription factor motif gene sets were used applying 1,000 permutations defined by the gene set. A weighed enrichment statistic using log2-ratio of classes was applied. A stringent limit with a nominal *P*-value < 0.001 and a FDR q-value < 0.01 was applied. In addition, we compiled a list of WNT target genes based on the WNT homepage [36] (see Additional file 1) and used a Yates corrected Chi-square test to compare our selected gene lists with the reference list. Other datasets were analyzed using a Mann-Whitney test for unpaired samples.

*In silico *promoter analysis of the *Col3a1, Col5a1 *and *Col5a3 *genes was performed using the TFSearch [37] and ALIBABA [38] online software, based on the TRANSFAC algorithm. Stringent criteria were applied so that only the responsive elements with a high homology to the consensus sequence matched our search (> 90%). Additionally, TCF/LEF responsive elements, specific transcription factors associated with WNT signaling, were investigated using the different consensus sequences as previously identified [39].

## Result

### Primary analysis of the microarrays

**Figure 1:**
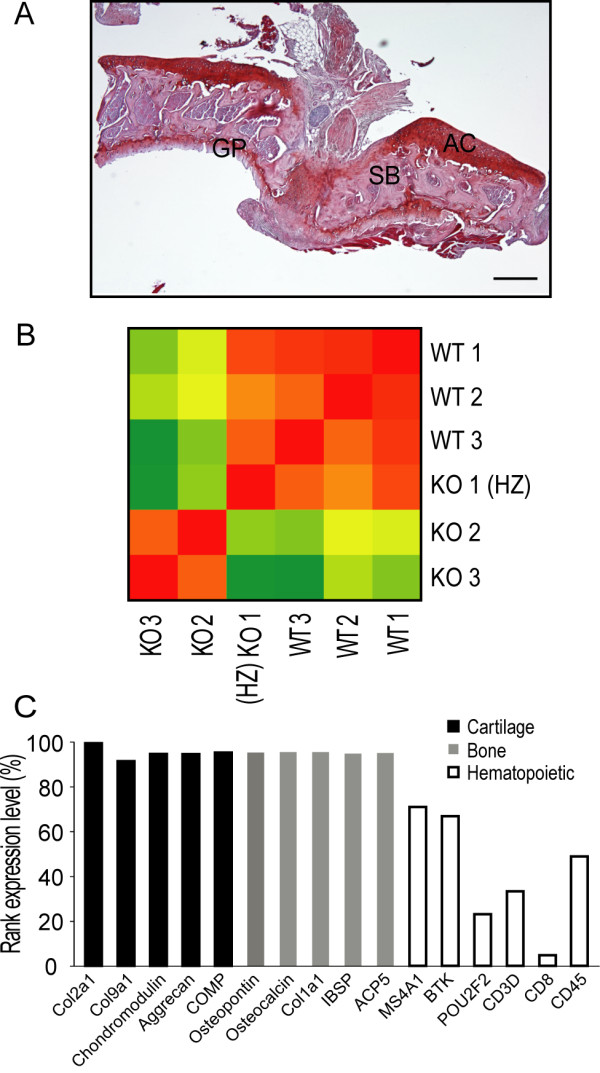
**Microarray analysis of cartilage and subchondral bone**. **(A) **Frontal hematoxylin-safranin O stained section of the tibia articular cartilage and subchondral bone isolated from a wild-type C57Bl/6 mouse at six weeks of age. Dissected tissues include the articular cartilage (AC), the underlying subchondral bone (SB) containing trabeculae and bone marrow and the upper part of the growth plate (GP). Scale bar = 50 μm **(B) **Heatmap showing the correlation between the Robust Multiarray Averaging (RMA) expression values for all samples. The three wild-type (WT) and one heterozygous frizzled-related protein (*Frzb^+/-^*) (HZ) mouse cluster apart from the two *Frzb^-/- ^*(KO) mice. Correlations are presented by colors going from green (lowest) to red (highest). **(C) **Analysis of the representation of genes in the microarray associated with articular cartilage (collagen type 2a1 (*Col2a1*), collagen type 9a1 (*Col9a1*), aggrecan, chondromodulin, cartilage oligomeric matrix protein (*Comp*)), bone (osteopontin, osteocalcin, collagen type 1a1 (*Col1a1*), bone sialoprotein 2 (*Ibsp*), tartrate-resistant acid phosphatase type 5 (*Acp5*)), and hematopoiesis (B-lymphocyte antigen CD20 (*Ms4a1*), B-cell progenitor kinase (*Btk*), lymphoid-restricted immunoglobulin octamer-binding protein (*Pou2f2*), CD3 antigen (*CD3*), CD8 antigen (*CD8*), protein tyrosine phosphatase, receptor type C (*CD45*)). Cartilage- and bone-specific genes were found in the highest percentiles, while T cell, B cell and platelet related genes, were found in lower amounts.

We were able to dissect the subchondral bone and articular cartilage in one piece (Figure 1A). The heatmap of the RMA expression values from the microarray analysis showed clustering of the transcriptomes into groups formed by the three wild-type and two out of three *Frzb*^-/- ^mice, respectively (Figure 1B). The third presumed *Frzb*^-/- ^mouse clustered with the wild-types and was subsequently identified by re-genotyping as a heterozygous animal. This sample was not used in the analysis. A total of 697 probe sets out of 30,590 that had a "present" detection call were significantly up-regulated in the *Frzb^-/- ^*samples and 1,524 were significantly down-regulated as compared to the wild-type mice (defined by a *P*-value < 0.01 after Benjamini-Hochberg correction and |log2|-ratio > 1). Cartilage-specific and bone-specific genes were found in the highest percentiles of expressed genes in the microarray analysis, whereas genes specifically related to T cells, B cells and platelets were found in lower percentiles; possibly from RNA originating from the subchondral bone marrow (Figure 1C).

Using the PANTHER resource, 493 mapped genes were identified as up-regulated and 905 mapped genes were identified as down-regulated in *Frzb*^-/- ^mice. The 25 genes with the largest fold-difference between *Frzb*^-/- ^and wild-type mice are presented in Table 1. A complete list of all regulated genes and fold differences can be found in the additional materials (see Additional file 2).

**Table 1:** Top 25 differentially up- and down-regulated genes by log fold change (LogFC)

Up-regulated genes			Down-regulated genes		
**Symbol**	**Name**	**LogFC**	**Symbol**	**Name**	**LogFC**
*Dbp*	D site albumin promoter binding protein	3.551	*Olfm4*	olfactomedin 4	-4.248
*Pck1*	phosphoenolpyruvate carboxykinase 1, cytosolic	2.836	*Igkv15-103*	immunoglobulin kappa chain variable 15-103	-4.238
*Rbm45*	RNA binding motif protein 45	2.689	*Apol11b*	apolipoprotein L 11b	-4.151
*Aspn*	asporin	2.464	*Frzb*	frizzled-related protein	-3.606
*Angptl7*	angiopoietin-like 7	2.461	*Mmp8*	matrix metallopeptidase 8	-3.531
*Htra4*	HtrA serine peptidase 4	2.442	*Cd5l*	CD5 antigen-like	-3.442
*Nnat*	neuronatin	2.367	*Slfn1*	schlafen 1	-3.388
*Epha3*	Eph receptor A3	2.367	*Apol8*	apolipoprotein L 8	-3.345
*Lrrn4cl*	LRRN4 C-terminal like	2.355	*Car1*	carbonic anhydrase 1	-3.338
*Angptl1*	angiopoietin-like 1	2.344	*Gypa*	glycophorin A	-3.328
*Matn4*	matrilin 4	2.315	*Spna1*	spectrin alpha 1	-3.294
*Olfml1*	olfactomedin-like 1	2.276	*Myb*	myeloblastosis oncogene	-3.264
*Col14a1*	collagen, type XIV, alpha 1	2.260	*Epb4.2*	erythrocyte protein band 4.2	-3.245
*Cdh13*	cadherin 13	2.230	*Ceacam10*	carcinoembryonic antigen-related cell adhesion molecule 10	-3.218
*Col14a1*	collagen, type XIV, alpha 1	2.202	*Spna1*	spectrin alpha 1	-3.175
*Sfrp1*	secreted frizzled-related protein 1	2.199	*Cd177*	CD177 antigen	-3.173
*Pck1*	phosphoenolpyruvate carboxykinase 1, cytosolic	2.189	*Igj*	immunoglobulin joining chain	-3.158
*Dkk3*	dickkopf homolog 3 (*Xenopus laevis*)	2.166	*Gypa*	glycophorin A	-3.156
*Nr1d2*	nuclear receptor subfamily 1, group D, member 2	2.142	*Rhd*	Rh blood group, D antigen	-3.152
*Adrb3*	adrenergic receptor, beta 3	2.138	*Fam55b*	family with sequence similarity 55, member B	-3.151
*Gnas*	guanine nucleotide binding protein, alpha stimulating	2.132	*Abca13*	ATP-binding cassette, sub-family A (ABC1), member 13	-3.142
*Dkk2*	dickkopf homolog 2 (*Xenopus laevis*)	2.131	*Mylk3*	myosin light chain kinase 3	-3.139
*Hmcn1*	hemicentin 1	2.116	*Ankrd43*	ankyrin repeat domain 43	-3.135
*Gpr1*	G protein-coupled receptor 1	2.115	*Ifitm6*	interferon induced transmembrane protein 6	-3.128
*Lrrc15*	leucine rich repeat containing 15	2.110	*Myb*	myeloblastosis oncogene	-3.111

### Pathway analysis

Different bioinformatics tools were used for analysis of the large dataset with emphasis on the identification of pathways differentially regulated between the *Frzb*^-/- ^and wild-type mice. The PANTHER pathway analysis is shown in Table 2. Among the up-regulated pathways the ECM-associated integrin pathway, the cadherin pathway, as well as WNT signaling, were most striking from a biological perspective. Down-regulated pathways pointed towards inflammation and immune cascades, the cell cycle, p53 activation and again integrins (Table 2). Associations of the differentially regulated gene set using databases defining "biological processes" as analysed by PANTHER are shown in the additional materials (see Additional file 3).

**Table 2:** PANTHER analysis of differentially expressed genes by pathway.

Pathway	number of genes in reference list	number of regulated genes	Expected number of genes	*P*-value*
**Pathways that are overrepresented taking up-regulated genes into account**				
Integrin signaling pathway	185	19	3.48	4.78E-09
WNT signaling pathway	348	22	6.55	1.27E-06
Angiogenesis	193	12	3.63	3.76E-04
Cadherin signaling pathway	167	11	3.14	4.07E-04
Alzheimer disease-presenilin pathway	124	9	2.33	6.84E-04
**Pathways that are overrepresented taking downregulated genes into account**				
B cell activation	87	21	3.01	1.03E-11
p53 pathway	127	18	4.39	8.32E-07
Inflammation mediated by chemokine and cytokine signaling pathway	298	29	10.30	1.12E-06
Blood coagulation	55	10	1.90	2.96E-05
p53 pathway feedback loops 2	51	9	1.76	9.15E-05
Parkinson disease	106	13	3.66	1.13E-04
Cell cycle	24	6	0.83	2.21E-04
Integrin signaling pathway	185	17	6.39	3.38E-04
*De novo *pyrimidine deoxyribonucleotide biosynthesis	23	5	0.79	1.36E-03
DNA replication	25	5	0.86	1.95E-03
Ras Pathway	80	9	2.76	2.20E-03
Apoptosis signaling pathway	141	12	4.87	4.37E-03
T cell activation	142	12	4.91	4.61E-03
JAK/STAT signaling pathway	20	4	0.69	5.48E-03
Angiogenesis	193	14	6.67	8.51E-03
**Pathways that are overrepresented taking up- and down-regulated genes into account**				
Integrin signaling pathway	185	36	9.87	7.87E-09
B cell activation	87	24	4.64	7.10E-09
Inflammation mediated by chemokine and cytokine signaling pathway	298	41	15.90	2.42E-06
p53 pathway	127	21	6.78	1.79E-04
Angiogenesis	193	26	10.30	4.45E-04
Blood coagulation	55	12	2.93	8.00E-04
p53 pathway feedback loops 2	51	9	2.72	2.42E-02
Cell cycle	24	6	1.28	2.19E-02
WNT signaling pathway	348	31	18.57	4.57E-02
Axon guidance mediated by netrin	29	6	1.55	4.36E-02
Parkinson disease	106	13	5.66	4.22E-02

* *P*-values are obtained by the binomial test; only *P*-values that fulfilled an additional false discovery rate test are shown.

We also applied the DAVID bioinformatics tools specifically interrogating gene representation in KEGG and Biocarta databases. Again, pathways associated with WNT signaling, cell adhesion and ECM interactions were most prominent among the up-regulated gene sets and appeared relevant from a biological perspective (see Additional file 4). Members of transforming growth factor-beta (TGFβ) superfamily signaling, including bone morphogenetic proteins (BMPs), were also up-regulated. Pathways among the down-regulated gene list were again linked to p53 signaling and the cell cycle, and to different systems associated with immunity and inflammation. The GSEA analysis further confirmed positive associations between *Frzb*^-/- ^mice and ECM interactions as well as negative associations with the cell cycle (see Additional file 5). No miRNAs were associated with the *Frzb^-/- ^*or wild-type phenotype using the stringent limit (nominal *P*-value < 0.001 and FDR q-value < 0.01). Only miRNA-147 had a nominal *P*-value < 0.001 and a FDR q-value < 0.25 (0.17). This miRNA has been associated with WNT and ECM pathways [40] (Table 3). In the transcription factor analysis, motifs associated with *Foxd1, Znf238 *and *Pbx1 *had nominal *P*-values < 0.001 and FDR q-values < 0.05. *Foxd1 *has been suggested as a WNT target gene in the developing chick retina [41] (Table 3). In addition, two motifs without specific transcription factor association were also enriched with *P*-values < 0.001 and FDR q-values < 0.05 (Table 3). Genes overexpressed in the wild-type mice compared to the *Frzb^-/- ^*mice were associated with different members of the E2F family of transcription factors applying the stringent criteria. E2F1 has been negatively associated with WNT signaling [42].

**Table 3:** miRNAs and transcription factors motifs for genes up-regulated in the *Frzb^-/- ^*mice

Name/Responsive Element sequence	Description	Enrichment Score	Nominal *P*-value	FDR* q-value
**miRNA associations in *Frzb^-/- ^*samples**				
miR-147	microRNA-147	0.49	0.000	0.172
**Transcription factor associations in *Frzb^-/- ^*samples**				
FoxD1	forkhead box D1	0.48	0.000	0.027
Znf238	zinc finger protein 238	0.45	0.000	0.018
Pbx1	pre B-cell leukemia transcription factor 1	0.49	0.000	0.014
RYTAAWNNNTGAY	No matching TCF	0.58	0.000	0.037
AAANWWTGC	No matching TCF	0.43	0.000	0.018

*False discovery rate (FDR)

### Detailed pathway analysis

We focused on a detailed analysis of changes in the WNT, the integrin/cadherin/ECM and the cell cycle pathways. Many genes mapped in the down-regulated inflammation-associated signaling systems were specifically linked to immune cell populations present in the bone marrow and were not further taken into account for this study.

The WNT pathway gene set demonstrated up-regulation of different extracellullar WNT antagonists in the *Frzb*^-/- ^mice as compared to wild-types. These genes belonged to the SFRP/FRZB-family, to the DKK family and to a group of intracellular WNT pathway modulators (Table 4 - compiled from PANTHER and DAVID analysis). Different frizzled (FZD) receptors were up-regulated and there was evidence for activation of both canonical and non-canonical signaling with increased expression of target genes, such as *Rspo2, Wisp2, Sox17, Tbl1x *and *Acta2*, and of intracellular messenger molecules *Nfatc2 *and *4 *that are activated in the calcium-dependent WNT pathway (Table 4).

**Table 4:** Genes linked to WNT signaling that are significantly up- or down-regulated

Symbol	Name	Function
**Up-regulated genes in *Frzb^-/- ^*mice**		
**WNT target genes**		
*Acta2*	actin, alpha 2, smooth muscle, aorta	Target gene
*Rspo2*	R-spondin 2 homolog (*Xenopus laevis*)	Target gene and stimulates WNT/β-catenin pathway
*Sox17*	SRY-box containing gene 17	Target gene
*Tbl1x*	transducin (beta)-like 1X-linked	Target gene and adaptor with the ubiquitin-conjugating/19S proteasome
*Wisp2*	WNT1 inducible signaling pathway protein 2	Target gene
**WNT antagonists**		
*Dkk2*	dickkopf homolog 2 (*Xenopus laevis*)	Extracellular WNT antagonist
*Dkk3*	dickkopf homolog 3 (*Xenopus laevis*)	Extracellular WNT antagonist
*Nkd1*	naked cuticle 1 homolog (*Drosophila*)	Intracellular antagonist
*Nkd2*	naked cuticle 2 homolog (*Drosophila*)	Intracellular antagonist
*Sfrp1*	secreted frizzled-related protein 1	Extracellular WNT antagonist
*Sfrp2*	secreted frizzled-related protein 2	Extracellular WNT antagonist
*Sfrp4*	secreted frizzled-related protein 4	Extracellular WNT antagonist
**WNT receptor**		
*Fzd1*	frizzled homolog 1 (*Drosophila*)	WNT receptor
*Fzd2*	frizzled homolog 2 (*Drosophila*)	WNT receptor
*Fzd8*	frizzled homolog 8 (*Drosophila*)	WNT receptor
**Intracellular messenger molecules**		
*Nfatc2*	nuclear factor of activated T-cells, cytoplasmic, calcineurin-dependent 2	Intracellular messenger in non-canonical calcium-dependent pathway
*Nfatc4*	nuclear factor of activated T-cells, cytoplasmic, calcineurin-dependent 4	Intracellular messenger in non-canonical calcium-dependent pathway
*Prickl2*	prickle homolog 2 (*Drosophila*)	Intracellular messenger in non-canonical planar cell polarity pathway
**Receptors of the TGFβ superfamily pathway**		
*AcvrI*	activin A receptor, type I	type I receptor for the TGFβ family
*AcvrIc*	activin A receptor, type IC	type I receptor for the TGFβ family
*BmprIb*	bone morphogenetic protein receptor, type 1B	type I receptor for the TGFβ family
**Cell adhesion molecules**		
*Cdh13*	cadherin 13	Cell adhesion molecule
*Dchs1*	dachsous homolog 1 (*Drosophila*) - protocadherin 16	Cell adhesion molecule
*Pcdh9*	protocadherin 9	Cell adhesion molecule
*Pcdh17*	protocadherin 17	Cell adhesion molecule
*Pcdh18*	protocadherin 18	Cell adhesion molecule
*Pcdh19*	protocadherin 19	Cell adhesion molecule
**Down-regulated genes in *Frzb^-/- ^*mice**		
**WNT antagonists**		
*Dkk1*	dickkopf homolog 1 (*Xenopus laevis*)	Extracellular WNT antagonist
*Btrc*	beta-transducin repeat containing	Stimulates β-catenin ubiquitinilation
**WNT receptor scaffolds**		
*Arrb1*	beta-arrestin 1	scaffold in intracellular WNT receptor complex
*Arrb2*	Beta-arrestin 2	

**Figure 2:**
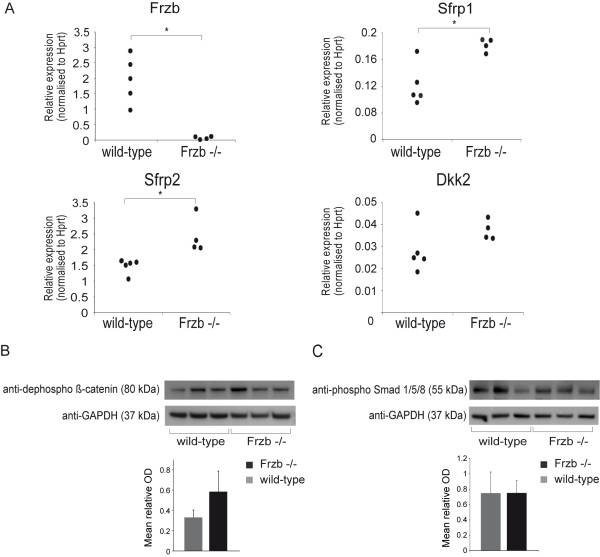
**Molecular analysis of the articular cartilage - subchondral bone to corroborate the microarray data**. **(A) **Real-Time PCR analysis of tibia articular cartilage and subchondral bone from frizzled-related protein-knockout (*Frzb^-/-^*) mice compared to wild-types. *Frzb *was virtually absent and secreted frizzled-related protein 1 (*Sfrp1*) and secreted frizzled-related protein 2 (*Sfrp2*) were significantly upregulated in *Frzb^-/- ^*samples compared to wild-type samples. There was a trend of up-regulation for dickkopf homolog 2 (*Dkk2*) (one outlier). Data are shown as relative expression values versus hypoxanthine guanine phosphoribosyl transferase (*Hprt*) (2^-ΔCt^) (*n *= four *Frzb^-/- ^*and five wild-type samples; All experiments were performed in duplicate; Mann-Whitney test: *P *= 0.016 for *Frzb, P *= 0.032 for *Sfrp1, P *= 0.016 for *Sfrp2 *and *P *= 0.2 for *Dkk2*). **(B-C) **Western blot and densitometry analysis of proteins extracted from tibia articular cartilage and subchondral bone showed no consistent change in active (dephospho) β-catenin (80 kDa) **(B) **and phosphorylated (phospho) Smad 1/5/8 (mothers against decapentaplegic homolog) (55 kDa) **(C) **between three wild-type (lane 1-3) and in three *Frzb^-/- ^*(lane 4-6) samples. Anti-GAPDH (glyceraldehyde-3-phosphate dehydrogenase) (37 kDa) Western blot is shown as loading control. Quantitative analysis was performed with Image J software. Data are shown as the ratio of the mean optical density (OD) for β-catenin or P-Smad/the mean OD of GAPDH (*n *= three samples/group; Mann-Whitney test: *P *> 0.05 for β-catenin and for P-Smad).

Confirmation experiments by RT-PCR showed lack of *Frzb*, significant up-regulation of *Sfrp1, Sfrp2 *and a similar trend for *Dkk2 *(Figure 2A). This up-regulation of other antagonists may represent a compensatory mechanism to minimise the effects of WNT pathway activation in *Frzb*^-/- ^mice. Western blot analysis showed only discrete amounts of these different antagonists in the dissected material and did not allow for reliable quantification of the individual proteins (data not shown). Baseline activation of the canonical signaling pathway was indeed not found different between *Frzb*^-/- ^and wild-type mice as demonstrated by Western blot and quantitative analysis by densitometry for the active form of β-catenin (Figure 2B). Also, Western blot for intracellular messengers of the BMP pathway, P-Smad 1/5/8, showed no striking differences between wild-type and *Frzb*^-/- ^mice suggesting maintenance of WNT and BMP pathway balance at the tissue level in unchallenged mice (Figure 2C). However, further comparison of the list with genes up-regulated in the *Frzb*^-/- ^mice with a user-compiled list of WNT target genes (see Additional file 1), did reveal consistent up-regulation of such targets indicating that more subtle changes at the molecular level are present (Yates corrected Chi-square test *P *< 0.0001).

Although we did not previously find structural abnormalities or spontaneous development of OA in *Frzb*^-/- ^mice, expression of ECM components and cell adhesion molecules showed a shift in this genetic model (Table 5). In particular, a number of collagens were differentially regulated and specific changes in integrins were found. Some of these link to the articular cartilage while others are more likely associated with the subchondral bone and with small vessels.

**Table 5:** Genes related to ECM matrix and cell adhesion

Symbol	Name	Function
**Up-regulated genes in *Frzb^-/- ^*mice**		
**Collagens**		
*Col3a1*	Collagen alpha-1(III) chain	Fibril forming collagen - co-distributes with type I collagen
*Col4a1*	Collagen alpha-1(IV) chain	Basal lamina collagen
*Col4a2*	Collagen alpha-2(IV) chain	Basal lamina collagen
*Col4a4*	Collagen alpha-4(IV) chain	Basal lamina collagen
*Col4a5*	Collagen alpha-5(IV) chain	Basal lamina collagen
*Col4a6*	Collagen alpha-6(IV) chain	Basal lamina collagen
*Col5a1*	Collagen alpha-1(V) chain	Fibril forming collagen - co-distributes with type I collagen
*Col5a3*	Collagen alpha-3(V) chain	Fibril forming collagen - co-distributes with type I collagen Type V collagen
*Col8a2*	Collagen alpha-2(VIII) chain	Basal lamina collagen
*Col12a1*	Collagen alpha-1(XII) chain	FACIT collagen
*Col14a1*	Collagen alpha-1(XIV) chain	FACIT collagen
*Col15a1*	Collagen alpha-1(XVIII) chain	Antiangiogenic factor - pericellular matrix
*Col16a1*	Collagen alpha-1(XVI) chain	FACIT-collagen
*Col18a1*	Collagen alpha-1(XVIII) chain	antiangiogenic factor - pericellular matrix
**Integrins**		
*Itga8*	Integrin alpha-8 light chain	RGD-binding integrin
*Itgbl1*	Integrin beta-like protein 1	Type B like integrin
*Sdc2*	syndecan 2	Heparan proteoglycan transmembrane protein
**Growth factors and receptors**		
*Egfr*	epidermal growth factor receptor	Receptor for epidermal growth factor
*Igf1*	insulin-like growth factor 1	Growth factor stimulating ECM synthesis
*Pdgfra*	platelet derived growth factor receptor alpha chain	Receptor for platelet derived growth factors
*Vegfc*	vascular endothelial growth factor c	Pro-angiogenic factor - Flt4 ligand
		
*Lims2*	LIM and senescent cell antigen-like-containing domain protein 2	Focal adhesion molecule
**Cytoskeleton molecules**		
*Acta2*	Actin, aortic smooth muscle	
*Parva*	Alpha-parvin	Actin binding protein
*Vcl*	Vinculin	Actin binding protein
**Extracellular matrix - integrin interacting molecules**		
*Lamb1*	laminin B1 subunit 1	Extracellular matrix glycoprotein
*Lamg1*	laminin, gamma 1	Extracellular matrix glycoprotein
*Npnt*	nephronectin	Ligand for integrin alpha 8/b1
*Tnn*	tenascin N	Extracellular matrix glycoprotein
*Tnxb*	tenascin XB	Extracellular matrix glycoprotein
*Thbs2*	thrombospondin 2	Extracellular matrix glycoprotein
*Thbs4*	thrombospondin 4	Extracellular matrix glycoprotein
**Down-regulated genes in *Frzb^-/- ^*mice**		
**Integrins**		
*Itga4*	Integrin alpha-4	
*Itgal*	Integrin alpha-L	
*Itgam*	Integrin alpha-M	
*Itgb2*	Integrin beta-2	
*Itgb2l*	Integrin beta-2-like protein	
**Focal adhesion molecule**		
*Lims1*	LIM and senescent cell antigen-like-containing domain protein 1	Focal adhesion molecule
**Cytoskeleton molecules**		
*Parvb*	Beta-parvin	Actin binding protein
Kinases		
*Map3k1*	Mitogen-activated protein kinase kinase kinase 1	Mitogen activated kinase
*Mapk13*	Mitogen-activated protein kinase 13	Mitogen activated kinase
*Grap2*	GRB2-related adaptor protein 2	Adapator protein
*Lck*	Proto-oncogene tyrosine-protein kinase LCK	T cell specific kinase
*Pik3cd*	Phosphatidylinositol-4,5-bisphosphate 3-kinase catalytic subunit delta isoform	Phosphoinositide 3-kinases
*Pik3cg*	Phosphatidylinositol-4,5-bisphosphate 3-kinase catalytic subunit gamma isoform	Phosphoinositide 3-kinases

**Figure 3:**
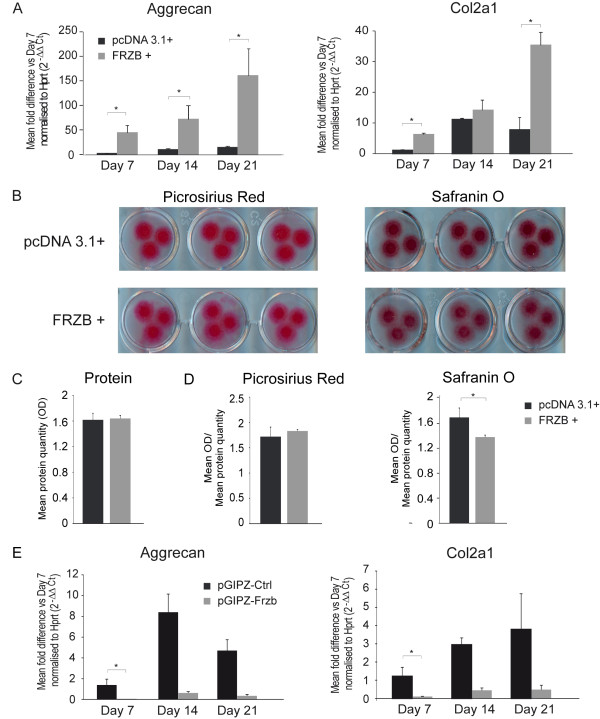
**Chondrogenesis after gain or loss of *FRZB *in ATDC5 cells**. **(A) **Real-Time PCR analysis showed increased expression of collagen type 2a1 (*Col2a1*) and *aggrecan *in the micro-masses overexpressing frizzled-related protein (*FRZB*) compared to micro-masses expressing control pcDNA3.1+ vector. Data are shown as the mean of the fold difference compared to the control condition at Day 7 normalised to hypoxanthine guanine phosphoribosyl transferase (*Hprt*) (2^-ΔΔCt^) ± SEM (*n *= six samples/condition; Mann-Whitney test: for *aggrecan P *= 0.002, *P *= 0.015 and *P *= 0.002 and for *Col2a1 P *= 0.03, *P *= 0.3 and *P *= 0.002). **(B) **Picrosirius Red and Safranin O staining at Day 7 showed increased spreading of collagen fibers and sulphated glycosaminoglycans (GAGs) from the center in micro-masses overexpressing *FRZB *compared to controls. **(C) **Protein quantification (optical density (OD) measured at 570 nm) of the micro-masses was comparable between the two groups (*n *= three samples/group; Mann-Whitney test: *P *> 0.05). **(D) **Staining intensity was comparable between *FRZB *overexpressing micro-masses and controls for Picrosirius Red and significantly decreased for *FRZB *overexpressing micro-masses for Safranin O staining. Data are shown as the mean OD normalised to the mean protein content (*n *= three samples/group; Mann-Whitney test: *P *> 0.05 for Picrosirius Red and *P *= 0.02 for Safranin O). **(E) **Real-Time PCR analysis showed decreased expression of *Col2a1 *and *aggrecan *in the micro-masses where *Frzb *was knocked down using the pGIPZ-shRNAmir directed against *Frzb *compared to controls. Data are shown as the mean of the fold difference compared to the control condition at Day 7 normalised to *Hprt *(2^-ΔΔCt^) ± SEM (*n *= two to three samples for pGIPZ-Ctrl and five to six samples for pGIPZ-FRZB; Mann-Whitney test: for *aggrecan P *= 0.02 and for *Col2a1 P *= 0.02). At Day 14 and Day 21 for pGIPZ-Ctrl *n *= 2 precluding statistical analysis.

**Figure 4:**
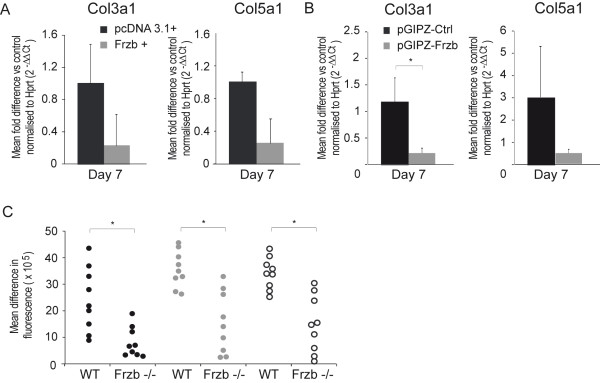
**Minor collagen expression after gain or loss of *FRZB *in ATDC5 cells**. **(A) **Real-Time PCR analysis for collagen type 3α1 (*Col3a1*) and collagen type 5α1 (*Col5a1*) expression in the ATDC5 micro-masses overexpressing frizzled-related protein *(FRZB) *compared to controls at Day 7. Data are shown as the mean fold difference compared to the control condition normalised to hypoxanthine guanine phosphoribosyl transferase (*Hprt*) (2^-ΔΔCt^) ± SEM (*n *= six samples/condition; Mann-Whitney test: for *Col3a1 P *= 0.24 and for *Col5a1 P *= 0.06). **(B) **RT-PCR analysis for *Col3a1 *and *Col5a1 *expression in the ATDC5 micro-masses where *Frzb *was knocked down using the pGIPZ-shRNAmir directed against *Frzb *compared to controls at Day 7. Data are shown as the mean fold difference compared to the control condition normalised to *Hprt *(2^-ΔΔCt^) ± SEM (*n *= six samples for pGIPZ-Ctrl and three samples for pGIPZ-FRZB; Mann-Whitney test: for *Col3a1 P *= 0.047 and for *Col5a1 P *= 0.54). **(C) **Proliferation assay of ribcage articular chondrocytes isolated from *Frzb^-/- ^*compared to wild-type (WT) mice. Data are shown as the difference in fluorescence after 24 h and one week. (*n *= nine conditions/group; Initial cell densities were 500 (black dots), 2,000 (grey dots) and 4,000 (black circles) cells per well; Mann-Whitney test: *P *= 0.0019, *P *= 0.0012 and *P *= 0.0008).

We performed complementary gain of function experiments to test the effect of FRZB on chondrogenesis and ECM composition in micro-masses from the mouse chondrogenic ATDC5 cell line. Expression of both *Col2a1 *and *aggrecan *was significantly increased in ATDC5 micro-masses overexpressing *FRZB *as compared to controls (Figure 3A). Staining for collagen content (Picrosirius Red) and sulphated glycosaminoglycans (GAGs) (Safranin O) at Day 7 revealed some changes in the morphology of micro-masses overexpressing *FRZB*. Collagen fibers and sulphated GAG distribution in these micro-masses seemed to have spread out more from the center compared to the controls (Figure 3B). Protein quantification of the micro-masses was, however, comparable between the two groups suggesting that the appearance reflects increased migration of ATDC5 cells overexpressing *FRZB *(Figure 3C). Quantification of the stainings was not different between micro-masses overexpressing *FRZB *and controls for Picrosirius Red. For Safranin O staining intensity was mildly but significantly decreased in micro-masses overexpressing *FRZB *(Figure 3D). Conversely silencing of *Frzb *resulted in down-regulation of these genes (Figure 3E). RT-PCR analysis of other collagens, in particular *Col3a1 *and *Col5a1*, significantly up-regulated in the *Frzb^-/- ^*mice compared to wild-type mice in the microarray analysis, depicted a decreasing trend at Day 7 in *FRZB *overexpressing micro-masses compared to the control micro-masses; however, these comparisons did not reach statistical significance (Figure 4A). A similar down-regulation compared to controls was seen during differentiation after silencing of *Frzb *(Figure 4B), which can be explained by the lack of chondrogenesis. *In silico *promoter analysis of these collagens, including *Col5a3*, which was also significantly up-regulated in *Frzb^-/- ^*samples, indicated the presence of several TCF/LEF responsive elements known from literature [39] in each of the gene promoters matching at least 80% of the original sequence. Moreover, each promoter contained a unique 100% consensus sequence in the promoter region indicating a direct link by which FRZB could modulate transcription of these genes (Table 6). Further analysis also showed the presence of binding sites for other transcription factors linked to WNT signaling such as *Oct-1, EP300, Gata *and *AP-1*.

**Table 6:** TCF/LEF responsive elements (RE) in collagen promoters and matching percentage (%)

TCF/LEF RE sequence	*Col3a1*	*Col5a1*	*Col5a3*
TTCAAAG	1 × 100%	1 × 100%	1 × 85%
CTTTGTT		3 × 85%	1 × 85%
CCTTTGATC		3 × 78-80%	1 × 80%
CCTTTGAT		3 × 85%	1 × 87.5%
CCTTTGAA		3 × 85%	1 × 87.5%
ATCAAAG	1 × 85%	2 × 85%	1 × 100%

Among the down-regulated pathways and processes, effects on the cell cycle and partially overlapping p53 signaling were most striking (Table 7). Down-regulation of different cyclins and cyclin kinases as well as many other positive regulators of the cell cycle suggest inhibition of mitosis and cell proliferation. Ribcage chondrocytes derived from *Frzb^-/- ^*mice proliferated significantly less than those derived from the wild-type mice *in vitro *after one week, corroborating the effect of FRZB on chondrocyte proliferation (Figure 4C).

**Table 7:** Genes linked to the cell cycle that are significantly down-regulated

Symbol	Name	Function
**Cyclins and cyclin kinases**		
*Ccna2*	Cyclin a2	
*Ccnb1*	Cyclin b1	
*Ccnb2*	Cyclin b2	
*Ccnd3*	Cyclin d3	
*Ccne1*	Cyclin e1	
*Ccne2*	Cyclin e2	
*Cdk1*	Cyclin dependent kinase 1	
*Cdk2*	Cyclin dependent kinase 2	
**Checkpoint regulators**		
*Bub1*	budding uninhibited by benzimidazoles 1	Kinase in spindle checkpoint function
*Bub1b*	budding uninhibited by benzimidazoles 1b	Kinase in spindle checkpoint function
*Chek1*	CHK1 checkpoint homolog	Checkpoint regulator of cell cycle
*Chek2*	CHK2 checkpoint homolog	Checkpoint regulator of cell cycle
*Mad2l1*	mitotic arrest deficient, homolog-like 1	Mitotic spindle checkpoint
**Minichromosome complex**		
*Mcm2*	minichromosome maintenance deficient 2 mitotin	Regulator of cell cycle
*Mcm4*	minichromosome maintenance deficient 4	Regulator of cell cycle
*Mcm5*	minichromosome maintenance deficient 5	Regulator of cell cycle
*Mcm6*	minichromosome maintenance deficient 6	Regulator of cell cycle
*Mcm7*	minichromosome maintenance deficient 7	Regulator of cell cycle
**Transcription factors**		
*E2F2*	E2F transcription factor 2	Regulator of cell cycle
*Tpdp1*	Transcription factor DP1	Partner of E2F transcription factors
*Tfdp2*	Transcription factor DP1	Partner of E2F transcription factors
*Ttk*	Ttk protein kinase	Mitosis associated kinase
**Other cell cycle regulators**		
*Cdc6*	cell division cycle 6 homolog	Regulator of cell cycle
*Cdc20*	cell division cycle 20 homolog	Regulator of cell cycle
*Cdc25a*	cell division cycle 25a homolog	Regulator of cell cycle
*Cdc25b*	cell division cycle 25b homolog	Regulator of cell cycle
*Cdc45*	cell division cycle 45 homolog	Regulator of cell cycle
*Dbf4*	DBF4 homolog (*S. cerevisiae*)	Activator of S-phase kinase
*Espl1*	extra spindle poles-like 1	Cleavage of sister chromatids
*Fzr1*	fizzy/cell division cycle 20 related 1	Activation of the anaphase promoting complex during mitosis
*GADD45A*	Growth arrest and DNA damage inducible gene 45a	Regulator of DNA repair and inhibitor of the S phase
*Orc1*	origin recognition complex, subunit 1	Initiation of DNA replication
*Orc6*	origin recognition complex, subunit 6	Initiation of DNA replication
*Plk1*	Polo-like kinase 1	Promoter of mitosis
*Pcna*	proliferating cell nuclear antigen	Cofactor of DNA polymerase delta
*Rb1*	Retinoblastoma 1	Regulator of the cell cycle
*Rbl1*	Retinoblastoma like 1	Regulator of the cell cycle

## Discussion

Our transcriptome analysis of the bone-cartilage biomechanical unit of *Frzb^-/- ^*and wild-type mice provides evidence for tight regulation of WNT signaling, shifts in ECM component synthesis and alterations in cell proliferation and differentiation. FRZB is a secreted WNT antagonist, originally identified from a chondrogenic extract of bovine articular cartilage [17] and misexpression of *FRZB *in the chick limb inhibits chondrocyte hypertrophy [18]. Polymorphisms in the human *FRZB *gene have been associated with OA [3], although this link has been debated recently [43].

Here, absence of *Frzb *in the articular cartilage and subchondral bone induces a subtle increase in WNT signaling evident by up-regulation of several WNT target genes as demonstrated by pathway analysis and by comparison with a user-compiled list of WNT target genes. Absence of *Frzb *also results in the up-regulation of other SFRP family members and different WNT modulators, suggesting that compensatory mechanisms exist in order to tightly control WNT signaling in these tissues. We previously demonstrated that *Frzb^-/- ^*mice show increased articular cartilage damage in different induced models of OA, although we did not see signs of spontaneous accelerated OA development in one-year old mice [7]. This contrasts with more direct and radical changes in the WNT canonical cascade as both tissue-specific gain and loss of function of β-catenin, result in premature OA [8,9].

FRZB can modulate both canonical and non-canonical WNT signaling. New insights into the differential activation of these pathways in articular chondrocytes may help to further explain why deletion of a single antagonist induces only subtle changes as compared to the dramatic effects of β-catenin modulation. Distinct SFRPs do not bind different WNTs with similar affinities and their effect may depend on the cell type and interactions with other pathways [44]. Nalesso *et al. *demonstrated that low amounts of WNT ligand can activate non-canonical signaling whereas higher amounts activate the β-catenin mediated pathway [45]. Moreover, inhibition of either pathway can de-repress the alternative one. In their system, Wnt3a induced articular chondrocyte dedifferentiation by activating the non-canonical Ca^2+^/CaMKII pathway and stimulated proliferation by activating the canonical pathway.

The changes we detected are not limited to the articular cartilage. Increased WNT signaling in the subchondral bone can also contribute to OA development. In this context, local regulatory mechanisms may be different from tissue to tissue. *Frzb*^-/- ^mice appear to have normal subchondral bone but increased cortical bone thickness [7]. Also, anabolic responses in the cortical bone to cyclic loading are much greater in *Frzb*^-/- ^mice compared to wild-types [7].

Absence of FRZB resulted in shifts in collagens, integrins and cadherins. Among these, changes in type III and type V collagen are of interest. As articular cartilage matures and ages, collagen fibrils become thicker, the amount of types IX and XI collagens decreases relative to type II collagen [46], and these minor collagens are progressively replaced by type V collagen [47]. Type III collagen can be detected in small but significant amounts in articular cartilage of mature joints and is cross-linked to the surface of type II collagen [46]. Its presence is more prominent in OA [48,49]. The type III collagen content in articular cartilage tends to vary between individual joints, anatomical location and tissue microanatomy. It may also be dependent on the history of injuries and the wear and tear experienced by a normal joint [46]. Therefore, it seems likely that type III collagen is synthesised as a modifier of existing fibril networks in response to tissue and matrix damage [46]. Although no increased cartilage damage was found in unchallenged *Frzb^-/- ^*mice, the significant up-regulation of *Col5a1, Col5a3 *and *Col3a1 *in the articular cartilage and subchondral bone from *Frzb^-/- ^*mice, suggests increased damage and repair in the *Frzb*^-/- ^mice at the molecular level.

These observations were further corroborated by complementary experiments where *FRZB *was overexpressed in the ATDC5 *in vitro *chondrogenesis model. Under these conditions, expression of both *Col3a1 *and *Col5a1 *was decreased during chondrogenic differentiation, suggesting that either FRZB by itself, or by modulating WNT signaling, affects expression of these ECM molecules in different systems. The additional observation that silencing of *Frzb *also results in a decrease in these collagens can be explained by lack of chondrogenic differentiation in the latter system.

We also found that overexpression of *FRZB *appeared to stimulate chondrogenesis in this model, as shown by increased *aggrecan *and *col2a1 *expression. Matured aggrecan monomers in the cartilage are glycosylated macro-molecules in which the glycoconjugates are formed by sulphatation of GAG side chains on the core protein [50]. The amount of sulphated GAGs in the micro-masses, measured by Safranin O staining, was surprisingly decreased in *FRZB *overexpressing micro-masses. Although the differences we observed were limited, these results might suggest that *FRZB *overexpression in this system impairs the maturation of these aggrecan monomers, for instance, by a relative excess in substrate due to the higher expression levels. Staining for collagens by Picrosirius Red indicated no major differences in total collagen content in *FRZB *overexpressing micro-masses and controls. The observed spreading of the fibers from the center, however, which was also noted in the Safranin O staining, suggests that overexpression of *FRZB *could modify matrix distribution, possibly by increasing ATDC5 migration. All these results are in line with earlier observations on FRZB and chondrogenesis [17,18].

Collagen type III and V are also found in the bone, co-distributed in much lower quantities next to the main collagen component type I collagen. Type V collagen expression is regulated by TGFβ in osteoblasts during osteogenesis [51]. Since members of the TGFβ pathway are up-regulated in our *Frzb*^-/- ^samples, this may affect expression in the subchondral bone. Collagen type V is increased in some patients with brittle bone disease and in patients with osteogenesis imperfecta, where collagen type V likely interferes with the normal process of mineralization [52]. Similar results were found for collagen type III, suggesting a role for collagen type III and V in defects in maturation of the bone [53-57].

The responsive elements for TCF/LEF but also other transcription factors, related to WNT signaling, in the *Col3 *and *Col5 *promoters suggest a direct link with WNT signaling by which FRZB can influence the composition of the cartilage and subchondral bone ECM. On the other hand, considering the relatively mild effects on WNT signaling at the tissue level, our study also leaves open the possibility that FRZB has unexpected, more robust post-transcriptional or epigenomic effects in these tissues suggesting new directions for research [58].

Loss of *Frzb *resulted in a decrease of genes associated with cell cycle progression. Proliferation analysis of ribcage chondrocytes isolated from *Frzb^-/- ^*mice compared to those isolated from wild-type mice agreed with this observation. Canonical WNT signalling is known to promote cell cycle progression and proliferation through the up-regulation of target genes like c-myc and cyclin D, but also via regulation of the mitotic spindle apparatus [59]. This apparent discrepancy where *Frzb^-/- ^*chondrocytes proliferate slower instead of faster, may be dependent on the cell type, the differentiation state, the WNT ligand involved and antagonist interactions. Differences in activation of either canonical or alternative pathways may also play a role.

The analysis presented here has a number of limitations. In particular, the number of samples used in the microarray experiment is small. Extraction of high quality RNA, required for microarray, from the articular cartilage is quite challenging due to a low cell content, the cross-linked extracellular matrix and considerably high levels of RNA degradation [60]. From this perspective, less than one-third of the extractions yielded RNA of sufficient quality and quantity for the analysis. In addition, transcriptome analysis does not convey information about proteins and post-translational modifications.

## Conclusions

These data further support an important role for FRZB in the homeostasis of the joint, in particular in the articular cartilage-bone biomechanical unit. The molecular up-regulation of other antagonists of the WNT signalling cascade in the absence of *Frzb *and the similar activation of the β-catenin mediated cascade also provide evidence for the important homeostatic potential of the joint. From the clinical perspective, this should encourage the search for compounds that stimulate tissue homeostasis. Further analyses and future studies should focus on fine mapping of the interactions between WNTs, their receptors and antagonists, as well as modulating effects of the inhibitors on their own. These investigations appear necessary to better understand the complex biology of WNTs and SFRPs in the joint, thereby, more precisely defining therapeutic targets and strategies. Again, from the clinical perspective, our study suggests that WNT pathway modulators should be carefully selected and linked to specific activation or inhibition of intracellular cascades in order to predict their potential effects and toxicity.

## Abbreviations

Acta2: actin, alpha 2, smooth muscle, aorta; BMP: bone morphogenetic protein; CamKII: calcium/calmodulin-dependent protein kinase II; c-myc: v-myc myelocytomatosis viral oncogene homolog (avian); Col2a1/3a1/5a1/5a3: collagen type 2α1/3α1/5α1/5α3; DAVID: database for annotation: visualization and integrated discovery; DKK: dickkopf; DMEM: Dulbecco's modified Eagle's medium; DNAsel: deoxyribonuclease; DPBS: Dulbecco's phosphate buffered saline; ECM: extracellular matrix; FDR: false discovery rate; FRZB: frizzled-related protein; GAGs: glycosaminoglycans; GAPDH: glyceraldehyde-3-phosphate dehydrogenase; GSEA: gene set enrichment analysis; HPRT: hypoxanthine guanine phosphoribosyl transferase; KEGG: Kyoto encyclopedia of genes and genomes; LEF: lymphoid enhancer factor; LRP5/6: low-density lipoprotein receptor-related protein 5/6; MAS: microarray analysis suite; MES: 2-(N-morpholino)ethanesulfonic acid; Nfatc2/4: nuclear factor of activated T-cells: cytoplasmic: calcineurin-dependent 2/4; OA: osteoarthritis; OD: optical density; PANTHER: protein analysis through evolutionary relationships; P-SMAD: phosphorylated-mothers against decapentaplegic homolog; PVDF: polyvinylidene difluoride; RE: responsive element; RMA: robust multiarray averaging; Rspo2: R-spondin 2; RT-PCR: real-time polymerase chain reaction; SDS: sodium dodecyl sulphate; SFRP: secreted frizzled-related protein; Sox17: SRY-box containing gene 14; Tbl1x: transducin (beta)-like 1X-linked; TBS: Tris-buffered saline; TCF: T cell factor; TGFβ: transforming growth factor: beta; Wisp2: WNT1 inducible signaling pathway protein 1; WNT: wingless-type MMTV integration site family member

## Competing interests

The authors declare that they have no competing interests.

## Authors' contributions

LL carried out all the experiments except for the experiments with ATDC5 cells. Experiments with ATDC5 cells were performed by ST and FC. Analysis of the micro-array data was performed by RL and LL. Manuscript preparation was carried out by LL, RL and FC. All other authors were involved in the design of the study, interpretation of the data and revision of the manuscript. All authors read and approved the final manuscript.

## Supplementary Material

Additional file 1 **Compiled list of WNT target genes based on the WNT Homepage**. Click here for file

Additional file 2 **Complete list of all regulated genes and fold differences**. Click here for file

Additional file 3 **Associations of the differentially regulated gene set using databases defining "biological processes" as analyzed by PANTHER**. Click here for file

Additional file 4 **DAVID analysis of differentially expressed genes by pathway**. Click here for file

Additional file 5 **GSEA analysis of all expressed genes by KEGG pathway**. Click here for file
