# Supplementary material for: Tight regulation of wingless-type signaling in the articular cartilage - subchondral bone biomechanical unit: transcriptomics in Frzb-knockout mice
Source: Arthritis Res Ther. 2012 Jan 20;14(1):R16. doi: 10.1186/ar3695 (PMC3392806; doi:10.1186/ar3695)
Supplement: Additional file 4 — DAVID analysis of differentially expressed genes by pathway. [file ar3695-S4.PDF]

Additional Table 4:

DAVID analysis of differentially expressed genes by pathway.

Pathway	Fold Enrichment	*p*-value*	Corrected *p*-value**
**Pathways overrepresented taking upregulated genes into account**			
ECM-receptor interaction	6.91	5.37E-09	6.61E-07
Focal adhesion	3.62	1.80E-06	1.11E-04
Axon guidance	4.11	1.31E-05	5.38E-04
Pathways in cancer	2.55	6.99E-05	2.15E-03
Wnt signaling pathway	3.37	2.23E-04	5.46E-03
TGF-beta signaling pathway	4.12	6.20E-04	1.26E-02
**Pathways overrepresented taking downregulated genes into account**			
Cell cycle	5.25	4.66E-17	6.61E-15
DNA replication	9.34	4.51E-13	3.20E-11
Leukocyte transendothelial migration	3.81	1.75E-08	6.22E-07
Hematopoietic cell lineage	4.54	1.47E-08	6.95E-07
B cell receptor signaling pathway	4.31	2.00E-07	5.67E-06
Natural killer cell mediated cytotoxicity	3.13	8.48E-06	2.01E-04
Mismatch repair	7.43	1.23E-05	2.49E-04
Primary immunodeficiency	5.55	1.52E-05	2.71E-04
Progesterone-mediated oocyte maturation	3.42	4.80E-05	7.57E-04
p53 signaling pathway	3.68	7.61E-05	1.08E-03
Chemokine signaling pathway	2.39	1.35E-04	1.74E-03
Fc gamma R-mediated phagocytosis	2.96	2.54E-04	2.77E-03
Base excision repair	4.54	2.43E-04	2.88E-03
Homologous recombination	5.38	4.81E-04	4.87E-03
Porphyrin and chlorophyll metabolism	4.84	9.56E-04	9.02E-03
Oocyte meiosis	2.53	1.43E-03	1.26E-02
Fc epsilon RI signaling pathway	2.88	1.56E-03	1.30E-02
Nucleotide excision repair	3.80	2.02E-03	1.59E-02
Cell adhesion molecules (CAMs)	2.12	4.35E-03	4.87E-03
**Pathways overrepresented taking both up- and downregulated genes into account**			
Cell cycle	3.77	1.61E-13	2.59E-11
DNA replication	6.20	3.41E-10	2.74E-08
Hematopoietic cell lineage	3.44	1.62E-07	8.72E-06
Leukocyte transendothelial migration	2.94	2.49E-07	1.00E-05
ECM-receptor interaction	3.20	2.33E-06	7.50E-05
B cell receptor signaling pathway	3.16	4.95E-06	1.33E-04
p53 signaling pathway	2.97	1.17E-04	2.69E-03
Focal adhesion	2.01	1.57E-04	3.15E-03
Mismatch repair	4.93	2.34E-04	4.19E-03
Pathways in cancer	1.72	2.91E-04	4.68E-03
Natural killer cell mediated cytotoxicity	2.27	3.58E-04	5.22E-03
Primary immunodeficiency	3.68	4.87E-04	6.51E-03
Cytokine-cytokine receptor interaction	1.78	8.12E-04	1.00E-02
Fc gamma R-mediated phagocytosis	2.34	9.66E-04	1.11E-02
Progesterone-mediated oocyte maturation	2.41	1.39E-03	1.49E-02
Small cell lung cancer	2.27	3.75E-03	3.49E-02
Cell adhesion molecules (CAMs)	1.88	3.73E-03	3.69E-02
Base excision repair	3.01	4.53E-03	3.98E-02
Homologous recombination	3.57	5.25E-03	4.36E-02
Pancreatic cancer	2.34	5.53E-03	4.37E-02

* p-values are obtained by a modified Fisher's Exact test; ** p-values are corrected for multiple testing by applying a Benjamini-Hochberg False Discovery Rate algorithm.
